# Genetic predisposition in children with cancer – affected families' acceptance of Trio-WES

**DOI:** 10.1007/s00431-017-2997-6

**Published:** 2017-09-19

**Authors:** Triantafyllia Brozou, Julia Taeubner, Eunike Velleuer, Martin Dugas, Dagmar Wieczorek, Arndt Borkhardt, Michaela Kuhlen

**Affiliations:** 1 Department of Pediatric Oncology, Hematology and Clinical Immunology, University Children’s Hospital, Medical Faculty, Heinrich Heine University, Moorenstr. 5, 40225 Duesseldorf, Germany; 2 Institute of Medical Informatics, University of Muenster, Muenster, Germany; 3 Institute of Human Genetics, Medical Faculty, Heinrich Heine University, Duesseldorf, Germany

**Keywords:** Cancer predisposition syndrome, Children, Trio, Whole-exome sequencing

## Abstract

A considerable percentage of childhood cancers are due to cancer predisposition syndromes (CPS). The ratio of CPSs caused by inherited versus de novo germline mutations and the risk of recurrence in other children are unknown. We initiated a prospective study performing whole-exome sequencing (WES) of parent-child trios in children newly diagnosed with cancer. We initially aimed to determine the interest in and acceptance of trio WES among affected families and to systematically collect demographic, medical, and family history data to analyze whether these point to an underlying CPS. Between January 2015 and December 2016, 83 (88.3%) of 94 families participated; only 11 (11.7%) refused to participate. Five (6.0%) children presented with congenital malignancies and three (3.6%) with tumors with a high likelihood of an underlying CPS. Two (2.5%) families showed malignancies in family members < 18 years, 11 (13.8%) showed relatives < 45 years with cancer, 37 (46.3%) had a positive cancer history, and 14 (17.5%) families had > 1 relative with cancer.

*Conclusions*: Genetic testing in pediatric oncology is of great interest to the families, and the vast majority opts for investigation into potentially underlying CPSs. Trio sequencing provides unique insights into CPS in pediatric cancers and is increasingly becoming a common approach in modern oncology, and thus, trio sequencing needs also to be integrated routinely into the practice of pediatric oncology.

**Table:** 

**What is Known:** • *A considerable percentage of childhood cancers are due to cancer predisposition syndromes (CPS).*
**What is New:** • *Knowing about an underlying CPS and, thus, the risk of recurrence in other children is of great interest to affected families.*

**Electronic supplementary material:**

The online version of this article (10.1007/s00431-017-2997-6) contains supplementary material, which is available to authorized users.

## Introduction

The proportion of children and adolescents with cancer attributable to an underlying cancer predisposition syndrome (CPS) is still unclear. Recent research studies indicate that a considerable percentage of childhood cancers are due to CPSs (16.7% of non-central nervous system (CNS) solid tumors, 8.6% of CNS tumors, and 4.4% of leukemias) [20]. However, in the era of high-throughput sequencing, it might be supposed that new CPSs will be discovered and, thus, the identification of affected children and their families will presumably increase within the next decade [9]. In addition, the ratio of CPSs caused by inherited versus de novo germline mutations in cancer predisposition genes (CPGs) and, thus, the risk of recurrence in other children is almost completely unknown so far. For example, the number of inherited TP53 germline mutations causing Li-Fraumeni syndrome (LFS) is estimated to be as high as 75% [1].

Indeed, mutations in CPGs involved in the DNA repair machinery, including mismatch and double-strand break repair, might have immediate implications on clinical decisions. For instance, LFS patients are highly susceptible to radiation-induced tumorigenesis and alkylating chemotherapy and, thus, have an increased risk of developing secondary cancers [5, 7].

Whole-exome sequencing (WES) of parent-child trios has become a popular strategy to identify causative genetic variants in children with rare diseases [4, 10, 21]. However, it has not been routinely implemented in pediatric oncology as yet. A number of reports on children developing metachronous tumors and families with familial clustering of malignancies suggest that trio sequencing in pediatric oncology can identify underlying CPSs [3, 6, 14].

Consequently, we initiated a monocentric prospective study on CPSs in a cohort of children and adolescents with a newly diagnosed malignancy by trio sequencing of the affected children and their parents. The main objectives of our study were first, to determine the interest in and acceptance of comprehensive clinical and molecular genetic evaluation in a pediatric oncology and hematology department; second, to systematically collect detailed demographic, medical, and family history data from this pediatric cancer cohort to analyze whether these data point to underlying CPSs; and third, to assess the proportion of children affected by either a well-known or suspected underlying CPS including the distribution pattern of contributing CPGs.

## Patients and methods

Since January 1st, 2015, an ongoing research study titled “Germline mutations in children with cancer” has been prospectively evaluating children and their parents by WES to test for underlying CPSs. All children (aged 0–18 years) with any newly diagnosed malignancy who were treated at the Department of Pediatric Oncology, Hematology and Clinical Immunology of the University Children’s Hospital and their parents were eligible. Families whose children died before the informed consent process was completed were excluded from this analysis. No other exclusion criteria were defined.

### Informed consent process

Informed consent was obtained in a multi-step process. In an initial conversation, the child (wherever possible) and the child’s parents were informed about the diagnosis and the study by being provided with the study information. In a second step, a few days after the diagnosis, the family was notified about the study aims, benefits, and risks in more detail, including implications for the patient and the entire family, the possibility of incidental findings and variants of unknown significance (VUS), options and preferences regarding how results should be reported, and their “right not to know.” In a third step, remaining questions were addressed and written informed consent was obtained. If the family was still undecided about participating, they were given more time for consideration. Pre-test counseling was provided by a pediatrician with a certificate in genetic counseling for genetic testing in pediatrics, as stipulated in the German gene diagnostic law. In cases where the family did not speak sufficient German, the informed consent process was performed with the help of a professional translator.

### Medical history and three-generation pedigree

Demographic data and the child’s medical history of previous malignancies and pre-existing conditions were collected through means of a standardized in-depth interview by a pediatrician (Supplement). This interview collected information regarding pregnancy, delivery, postnatal adaptation, development during early childhood, congenital anomalies, and other specific symptoms. As references for the comparison of birth data and data on assisted conception, the Annual Report (2016) of the Federal Statistical Office (Destatis) and the Annual Book (2015) of the German IVF Registry (DIR) were used.

All patients were thoroughly examined by a pediatrician, with particular attention to congenital anomalies and signs or conditions suggesting an underlying syndrome. Additionally, information on tumor/leukemia features pointing to an underlying germline defect was recorded and excessive toxicity to cancer therapy was prospectively evaluated.

Three-generation pedigrees (patient, parents, grandparents, siblings, uncles, and aunts) were constructed for each participating family, including information on birth date, deceased, age at and cause of death, symptoms.

DNA for WES analysis was extracted pre-therapeutically either from peripheral blood in patients with solid tumors or from skin biopsies using fibroblasts in patients with leukemia or lymphoma. Peripheral-blood-derived DNA from the parents was used for WES.

**Fig. 1:**
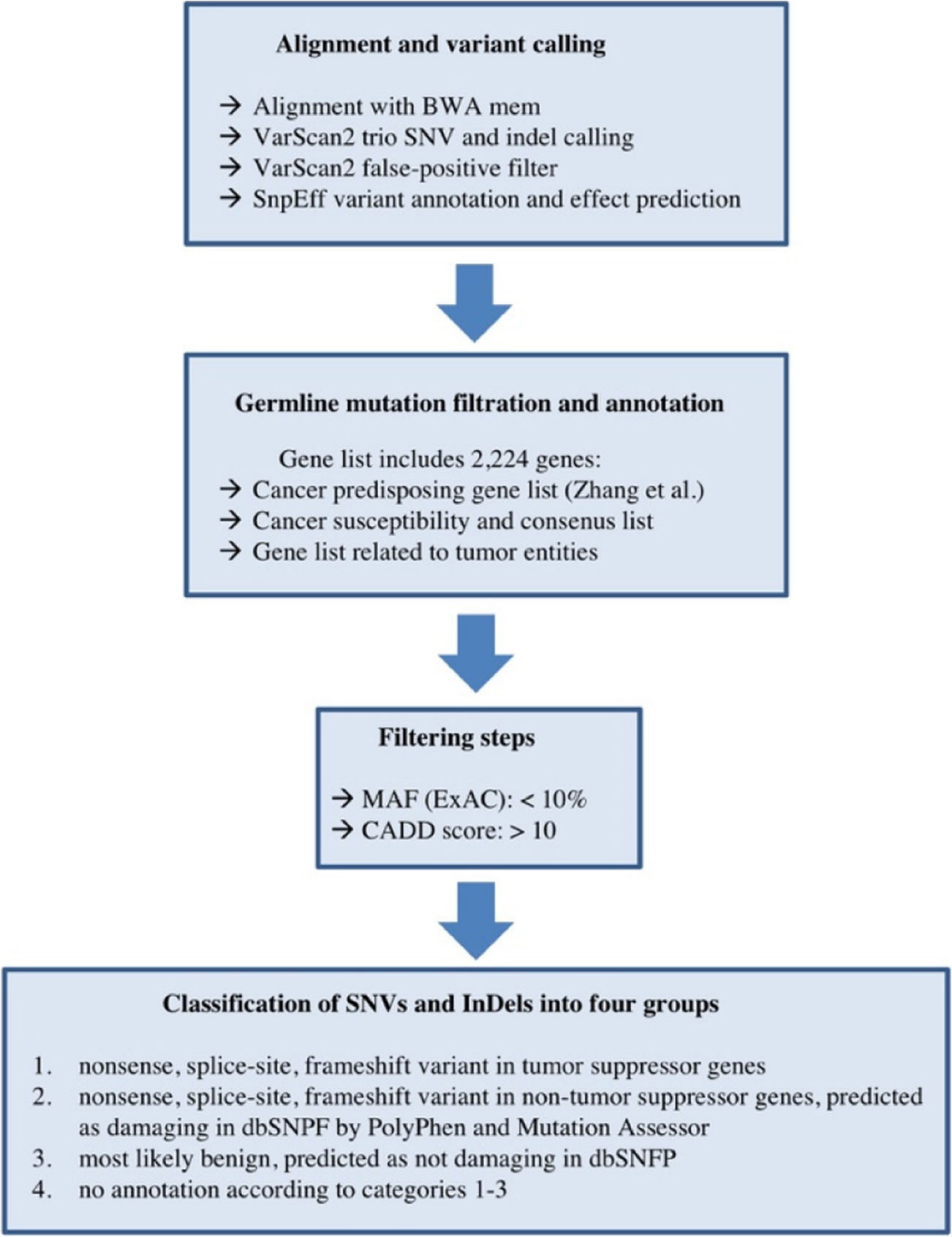
Overview of the bioinformatic pipeline

A bioinformatic pipeline was established based on data analysis published by the St. Judes study group and a constantly updated gene list currently comprising 2224 genes including the 565 known cancer-predisposing genes, which were summarized by Zhang et al. [20]. To identify only relevant single nucleotide variants (SNVs) by WES, we defined the following analysis criteria: (1) a high-quality DNA sequencing coverage of ≥ 250-fold (to additionally identify parental mosaicism); (2) variants with a minor allele frequency (MAF) below 10%; (3) SNVs in any of the 2224 genes of the cancer gene list with non-synonymous coding changes; (4) in silico prediction tools (SIFT and PolyPhen) considering the identified variant as (probably/possibly) damaging or deleterious for protein function; and (5) a CADD (combined annotation-dependent depletion) [8] score > 10. An overview of the bioinformatic pipeline is given in Fig. 1.

For this analysis, the data of patients enrolled between 1 January 2015, and 31 December 2016, were examined. The study was approved by the Ethics Committee of Heinrich Heine University, Duesseldorf, Germany (study number 4886).

## Results

Between 1 January 2015, and 31 December 2016, 94 families of children and adolescents with a newly diagnosed malignancy were asked to participate in the study. Of these, 83 (88.3%) families agreed to participate in the study and 11 (11.7%) families refused to participate. Reasons for refusal were fear of the results in six cases (four of them with a positive cancer history in the family), uncertainty and mental overload in three families, and cultural objections in two families (none of them with a positive familial cancer history). In one of these cases, the adolescent patient refused to participate (due to fear) while both parents wanted to participate.

**Fig. 2:**
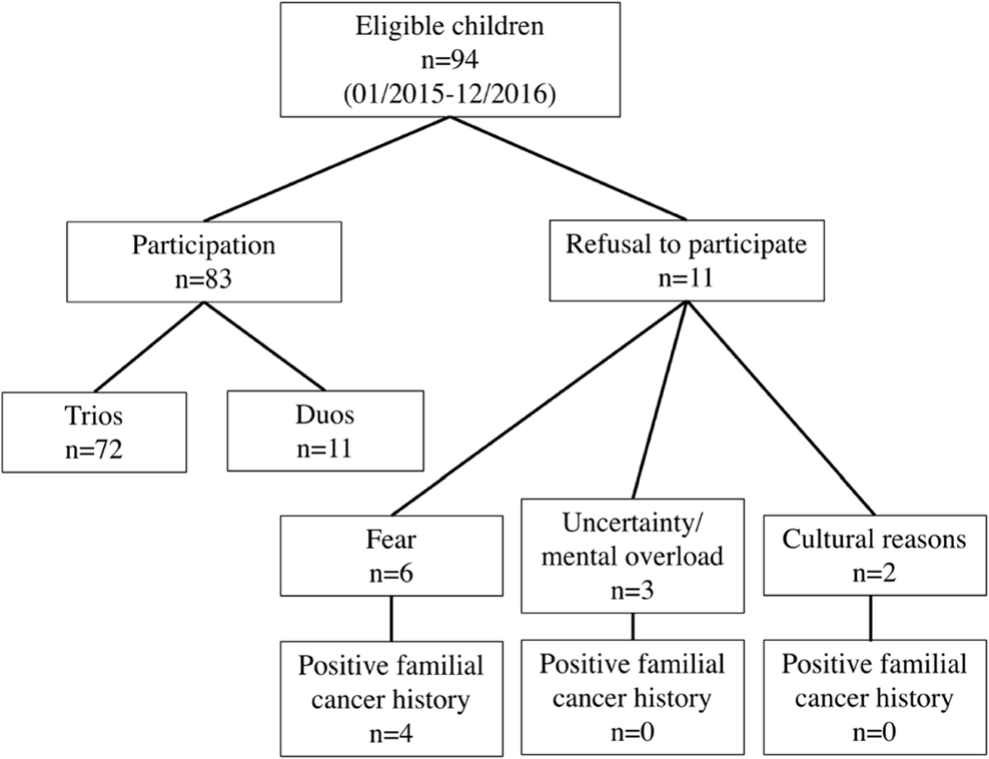
Overview on participation and refusal reasons of families with children with a newly diagnosed malignancy (*n* = 94)

In 11 families, only one parent was available, due to either a lack of contact information (nine cases) or one parent having already died (two cases, both due to cancer). In one family, consent to participate in the study was given by both parents for the child but one parent (with a highly suspicious familial cancer history) refused to be tested him-/herself. Thus, in these cases, only duo sequencing was feasible. Details on recruitment and refusal are depicted in Fig. 2.

### Patient characteristics and medical history

Demographic characteristics of participating families are listed in detail in Table 1. The mean number of children per family was 2.4 (range 1–6).

**Table 1:** Demographic characteristics of participating patients and their families (*n* = 83)

Gender	
Male	54 (65.1%)
Female	29 (34.9%)
Age at onset in years, median (range)	6.0 (birth–18.0 years)
Parental age in years, median (range)	
Father	34.3 (20.2–50.3)
Mother	29.9 (18.0–48.8)
Siblings, median (range)	
None	23 (27.7%)
1–2	47 (56.6%)
≥ 3	13 (15.7%)

In 80 (96.4%) of 83 families, complete details on medical history including birth date, cause of death, and, in the case of a positive cancer history, also the type of cancer were available for further analysis in a three-generation pedigree. In addition to the information provided by the families, in some cases we also asked for and received medical records of the affected family members. Of these 80 children, three (3.8%) children’s parents reported the use of assisted reproductive technologies (ART), three (3.8%) children presented with congenital heart defects, four (5.0%) children with café-au-lait spots, six (7.5%) children with pre-existing conditions (Asperger’s syndrome, attention deficit hyperactivity disorder, depression, strabismus, splenic cyst, and hemangioma), and two (2.5%) children with a history of a previous malignancy (further details are given in Table 2).

**Table 2:** Details on medical history of the patients (*n* = 80)

Assisted reproductive technology	
No	76 (95.0%)
Hormonal	1 (1.3%)
IVF/ICSI	3 (3.8%)
Abnormalities during pregnancy	
No	62 (77.5%)
Yes	18 (22.5%)
Small for gestational age	
No	75 (93.8%)
Yes	5 (6.3%)
Prematurely born	
No	72 (90.0%)
Yes	8 (10.0%)
Postpartal adaptation	
Regular	70 (87.5%)
Remarkable	10 (12.5%)
Development in early childhood	
Regular	70 (87.5%)
Remarkable	7 (8.8%)
Not applicable	3 (3.8%)
Congenital anomalies	
No	73 (91.3%)
Yes	7 (8.8%)
Pre-existing conditions other than congenital anomalies	
No	74 (92.5%)
Yes	6 (7.5%)
History of previous malignancies	
No	78 (97.5%)
Yes	2 (2.5%)

Comparing these data with data from the Federal Statistical Office and the German IVF Registry, no differences were observed in parental age, prematurity, or the number of reported ARTs used [16].

**Fig. 3:**
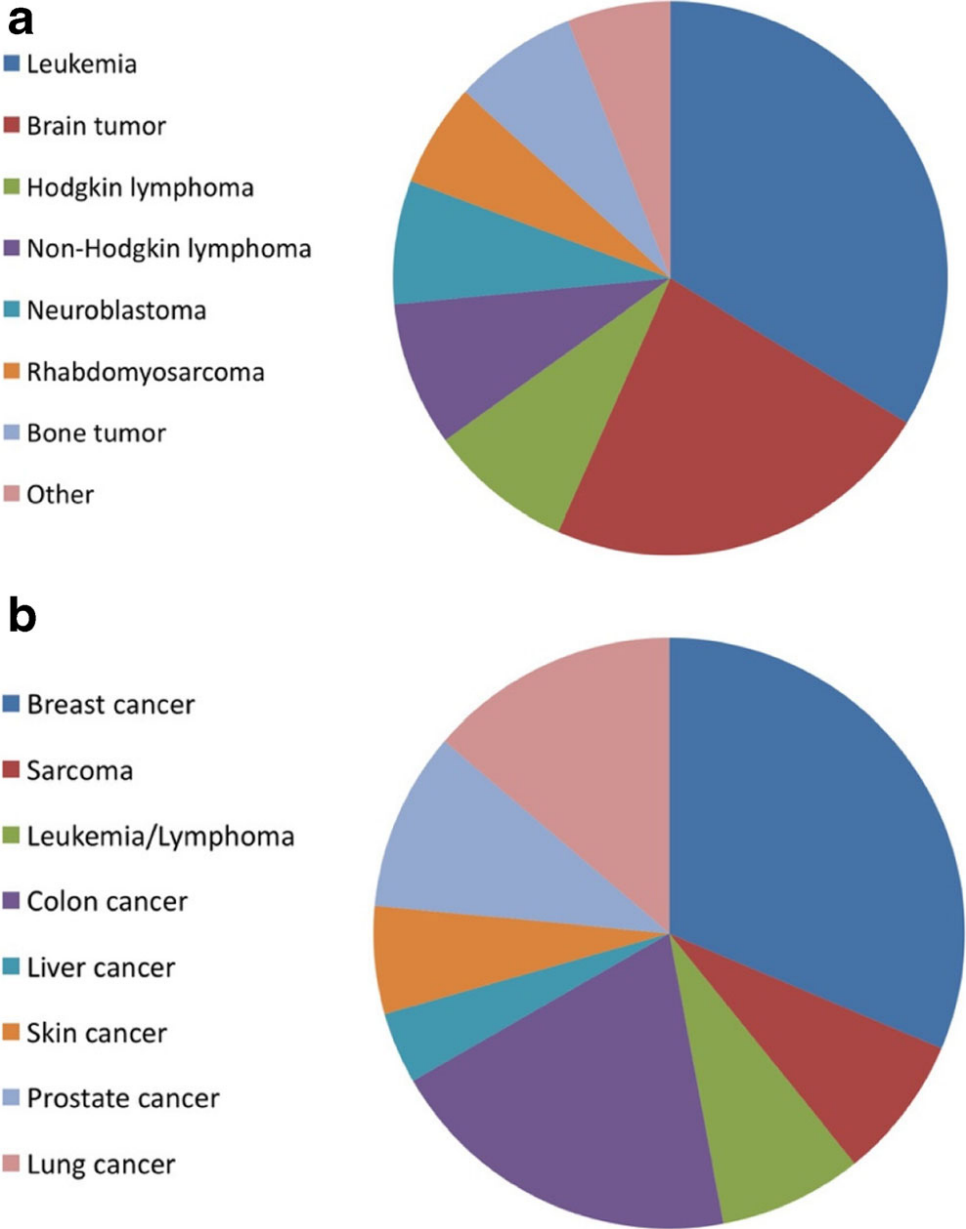
**a** Overview of the diagnoses of children with cancer enrolled in the study (*n* = 83). **b** Overview of cancer diagnoses in first- or second-degree relatives. Same cancer entities were counted just once per family

Of 83 children, most were diagnosed with leukemias (28, 33.7%) and brain tumors (19, 22.9%) (Fig. 3a). Three (3.6%) presented with tumors including hypodiploid ALL, plexus carcinoma, and pleuropulmonary blastoma (PPB) with a high likelihood of an underlying germline defect. Six (7.2%) children developed excessive toxicity (grade 4 mucositis, neurotoxicity, veno-occlusive disease, hyperammonemia, and grade 5 respiratory failure) to cancer therapy, which was either particularly long-lasting or developed in a therapy regimen normally not associated with that kind of toxicity according to expert clinical experience. In two of these children, a CPS was subsequently diagnosed. Five (6.0%) children presented with congenital leukemias or tumors (Table 3).

**Table 3:** Tumor specifics and cancer therapy tolerance in participating children and adolescents (*n* = 83)

Congenital tumor	
No	78 (94.0%)
Yes	5 (6.0%)
Tumor with high likelihood of germline defect	
No	79 (95.2%)
Yes	3 (3.6%)
Not applicable	1 (1.2%)
Excessive toxicity to cancer therapy	
No	74 (89.2%)
Yes	6 (7.2%)
Not applicable	3 (3.6%)

### Three-generation pedigree

Three-generation pedigrees revealed malignancies in family members under the age of 18 years in two (2.5%) of 80 families, relatives with cancer before the age of 45 years in 11 (13.8%) families, any cancer history in 37 (46.3%) families, and more than one relative with cancer in 14 (17.5%) families (Table 4). No parents were consanguineous. More precisely, first- or second-degree relatives presented with (mostly premenopausal) breast cancer (including one father) in 16 (20.0%) families, sarcoma in four (5.0%) families, leukemia/lymphoma in four (5.0%) families, and colon cancer in ten (12.5%) families. An overview of cancer diagnoses in first- or second-degree relatives is given in Fig. 3b.

**Table 4:** Details on three-generation pedigree (*n* = 80)

Malignancies in family members under the age of 18 years	2 (2.5%)
Relatives with cancer > 18–45 years of age	11 (13.8%)
≥ 2 first- or second-degree relatives in the same parental lineage with cancer under the age of 45 years	3 (3.8%)
Any cancer history	37 (46.3%)
More than 1 relative with cancer	14 (17.5%)
Deaths due to cancer	22 (27.5%)

### Reporting of results

In case of an underlying CPS, validation of the identified mutation was carried out by Sanger sequencing before the results were reported to the families. To exclude sample swap, confirmation of the mutation was performed using a second peripheral blood sample by Sanger sequencing.

The treating pediatrician told the parents when the results were ready. If the parents wanted to know the results, a member of the study team met with the parents and, wherever possible, with the child to explain and discuss the results. In total, 82 (98.8%) of 83 families wanted to be informed about the results, and none of them changed their minds when the results were available. Due to a positive family history and fear, one family did not want to be informed. When a CPS was identified, an appointment with a genetic counselor was recommended to the parents. If a CPS was then diagnosed, the affected child was integrated into a cancer surveillance program either according to published recommendations (for LFS) or individually conceptualized and subsequently adapted according to the recommendations of the Cancer Predisposition Workshop of the American Association of Cancer research (AACR) [11, 12]. Examples of children in whom diagnosis of a CPS led to adaptation of cancer therapy and/or inclusion in a cancer surveillance program, respectively, are given in Table 5.

**Table 5:** Examples of adaptation of cancer therapy and/or inclusion in a cancer surveillance program after diagnosis of a CPS by trio WES

CPS and type of cancer	Adaptation of cancer therapy	Cancer surveillance program according to
Li-Fraumeni syndrome in hypodiploid ALL	Omission of cranial irradiation; instead, administration of additional intrathecal chemotherapy	Kratz et al. Clin Canc Res (2017)
Li-Fraumeni syndrome in plexus carcinoma	Omission of cranial irradiation; instead, high-dose chemotherapy with autologous stem cell transplantation	Kratz et al. Clin Canc Res (2017)
Constitutional mismatch repair deficiency in medulloblastoma	None	Tabori et al. Clin Canc Res (2017)
Dicer syndrome in pleuropulmonary blastoma	None	Schultz et al. Clin Canc Res (2017)
Gorlin syndrome	None	Schultz et al. Clin Canc Res (2017)
Neurofibromatosis type I in glioma	Omission of cranial irradiation; instead, systemic chemotherapy	Evans et al. Clin Canc Res (2017)

As examples, brief descriptions of three families are given.

Case #1: The 8-month-old boy was diagnosed with plexus carcinoma, a rare tumor with a high likelihood of an underlying germline defect. The parents did not have other children. The family history was highly suggestive of an existing CPS, including three second-degree relatives with osteosarcoma diagnosed under the age of 45 years and premenopausal breast cancer in the paternal lineage. However, genetic counseling had not been performed so far. Trio WES analysis confirmed a heterozygous germline mutation in TP53 (p. Gly245Ser, c.733G>A), suggesting LFS, which is transmitted by the father and predicted to be pathogenic and disease causing. Response to international treatment recommendations (CPT SIOP 2009 including vincristine, cyclophosphamide, etoposide, doxorubicine, cisplatin, and actinomycin) was poor. Thus, normal radiotherapy was indicated. Due to an underlying LFS, an individual treatment concept omitting radiotherapy was created, which was based on drug resistance testing of the tumor (including bortezomib) and high-dose chemotherapy (including thiotepa, carboplatin, and etoposide) with autologous stem cell transplantation. On day + 120, the boy is well without evidence of relapse.

Case #2: The 13-month-old girl presented with desmoplastic medulloblastoma and skin features reminiscent of constitutional mismatch repair deficiency (CMMRD). The non-consanguineous parents (both under the age of 30 years) and an older sister were healthy, as were three generations of the family. Two inherited homozygous VUS of *MSH2* (c.274C>G, p.Leu92Val) and *MSH6* (c.2426_2428delTAG, p.Val809del) were identified by WES and, thus, further raised the suspicion of CMMRD. As a differential diagnosis, germline mutations in *POLD1* and *POLE* were ruled out [19]. Tumor microsatellite instability testing and immunohistochemistry analysis were inconclusive. Therefore, in collaboration with the CMMRD consortium [18], functional analyses were initiated to confirm diagnosis of CMMRD (Fremerey et al. submitted). The girl was treated according to the HIT guidance protocol without radiotherapy due to her young age. Two years onwards, the girl is well and in radiological complete remission.

Case #3: The 10.5-year-old boy was diagnosed with periosteal osteosarcoma. His medical history was remarkable, with embryonal rhabdomyosarcoma of the thoracic wall at the age of 1.75 years. At that time, treatment comprised alkylating agents but no radiotherapy. His parents and dizygotic twin brother were healthy. An uncle in the paternal lineage was deceased due to cancer (further details were not available). Applying the abovementioned criteria for single nucleotide variants (SNVs) analysis, we did not detect any SNV fulfilling these conditions.

## Discussion

Here, we evaluated the interest in and acceptance of comprehensive clinical and molecular genetic screening for an underlying CPS, including trio whole-exome sequencing of parents with a child diagnosed with cancer. Our data suggest that knowledge of an underlying CPS is of great interest to the families in our sample and that the vast majority of parents do not claim their right not to know. Instead, most families participated immediately, as they hoped to find a reasonable explanation for why their child had been struck with such an extraordinarily rare event in childhood and to learn about the risk of recurrence in their other children.

Whenever next generation sequencing (NGS) is initiated, according to the gene diagnostic law, the treating physician is obligated to discuss the full range of benefits, risks, and alternatives of this particular genetic test including the potential to reveal gene abnormalities related to other disorders. However, disclosing the diagnosis of cancer is overwhelming and dramatically limits the child’s/parents’ receptivity. Consequently, in the daily clinical routine of pediatric oncology, this leads to the imperative necessity for a time-consuming multi-step process as depicted above. Indeed, the decision-making process of the families sometimes takes a few months. In our sample, only a minority of families exercised their “right not to know”. These were either families with a highly suspicious familial cancer history or families who refused to participate due to their cultural background. This is in line with the empirical expert knowledge that parents frequently ask whether their other children have an increased risk of developing cancer.

Identifying children with a hereditary CPS by trio WES has far-reaching consequences that extend beyond providing cancer care for the child. Close and more distant relatives might likewise be affected despite being young and as-yet asymptomatic. Disclosing a hereditary CPS in these relatives might be clinically relevant and even lifesaving on the one hand, as it provides the excellent possibility to initiate early cancer surveillance programs [11]. On the other hand, it constitutes an enormous life-long burden of knowledge and might deeply affect quality of life and family planning. In this context, the potential advantages and drawbacks as well as personal autonomy regarding the “right not to know” must be discussed in detail before initiating trio WES. An appointment with a genetic counselor was strongly recommended to families in which an underlying CPS was diagnosed. However, a discussion about further genetic testing of additional family members must consider that, in contrast to standards for genetic testing in adults, predictive testing in children is recommended only when the disease is associated with childhood onset, and only with available effective screening and/or intervention options [2, 13]. Refraining from predictive testing in childhood allows the child to make this decision autonomously when reaching adulthood. As one child had already developed cancer, disease onset during childhood is given in all families. Nevertheless, cancer surveillance programs which are advantageous to survival exist only for LFS to date, but still need to be established and proved to be beneficial for other CPS.

We could not identify features in pregnancy, delivery, congenital anomalies, postnatal adaptation, or development during early childhood that pointed towards an underlying CPS. Moreover, neither parental age nor ART seems to be associated with an increased cancer risk in our study cohort. This is in line with previous findings that children born after ART are not at increased cancer risk [15, 17].

However, 3–8% of the children presented with suggestive clinical features (e.g., café-au-lait spots), tumors with a high likelihood of an underlying germline defect, or excessive toxicity to cancer therapy. In addition, in a remarkable number of families, the three-generation pedigree revealed a highly suggestive family history. Thus, although this is an observational study with respective limitations, our preliminary findings demonstrate that a thorough clinical examination and in-depth family history might point towards an underlying CPS, which is in contrast to previous findings by Zhang et al. [20].

However, a highly suggestive medical history, such as one including metachronous tumors, or an unremarkable family history could both be misleading. The latter is of particular importance, as the number of de novo TP53 germline mutations causing LFS is estimated to be as high as 25% [1]. Notably, the proportion of CPSs caused by de novo germline mutations in other CPGs is so far completely unknown.

Thus, our study provides a highly valuable resource to determine the type, frequency, and the de novo mutation rate of CPSs in a cohort of newly diagnosed pediatric cancer patients and may eventually identify novel CPSs in the future.

## Conclusions

In pediatric oncology, testing for an underlying CPS seems to be more important to the affected families than exercising their right of not knowing. In order to gain better insights into the ratio between inherited risk alleles found throughout the family and acquired de novo mutations, trio sequencing needs to be integrated routinely into the practice of pediatric oncology

## Electronic supplementary material

ESM 1 (DOCX 186 kb)

## Abbreviations

AACR: Association of Cancer Research
ART: Assisted reproductive technologies
BWS: Beckwith-Wiedemann syndrome
CMMRD: Constitutional mismatch repair deficiency
CNS: Central nervous system
CPG: Cancer predisposition gene
CPS: Cancer predisposition syndrome
LFS: Li-Fraumeni syndrome
MAF: Minor allele frequency
NGS: Next generation sequencing
VUS: Variant of unknown significance
WES: Whole-exome sequencing
