# Supplementary material for: Genetic predisposition in children with cancer – affected families' acceptance of Trio-WES
Source: Eur J Pediatr. 2017 Sep 19;177(1):53–60. doi: 10.1007/s00431-017-2997-6 (PMC5748429; doi:10.1007/s00431-017-2997-6)
Supplement: Supplementary file 1 [file 431_2017_2997_MOESM1_ESM.docx]

ANAMNESEBOGEN DATUM: ____________

Name:__________________ Vorname:________________ Geb.-Datum:___________________

Geschlecht: ☐ M ☐ W Alter: ____ J ____M

Künstliche Befruchtung

☐ keine ☐ hormonelle Stimulation ☐ Insemination ☐ IVF ☐ ICSI

Verlauf der Schwangerschaft

☐ *ungestört*

☐ Kindsbewegungen____

☐ Pränataldiagnostik, wann, wo?_________________________________

☐ Blutungen/drohende Fehlgeburt/vorzeitige Wehen?____________________

☐ Alkohol-/Drogenkonsum/Medikamenteneinnahme_________________________

☐ Erkrankungen____

☐ Sonstiges____

Geburt in der ___. SSW

Geburtsgewicht:________________ Länge: ______cm Kopfumfang:_______cm

APGAR:_____/_____/________ Nabelschnur-pH:__________

Geburtsverlauf: spontan / Sektio / Zange / Saugglocke

☐ sich verschlechterndes CTG? ☐ grünes Fruchtwasser?

☐ *problemlos*

☐ Auffälligkeiten?____

☐ Behinderte Atmung, Atemhilfe, Beatmung?__________________________

☐ Inkubator?

☐ Herzfehler?____

Postpartale Entwicklung

☐ *unkompliziert*

☐ Ikterus?

☐ Trinkschwierigkeiten?

☐ Muskeltonus ☐ hypoton ☐ hyperton ☐ unauffällig

☐ Kopfkontrolle?____

☐ Krankengymnastik ☐ Ergotherapie ☐ Frühförderung ☐ Logopädie?

☐ Sitzen:____________ Krabbeln:____________ Laufen:____________

☐ Zahnentwicklung____________

☐ Sprachentwicklung____________

☐ Sprachverständnis____________

☐ Sauberkeitserziehung____________

☐ Aggression? Unruhe?____________

☐ Schwere Erkrankungen? Gehäufte Infekte?____________

☐ Operationen?____________

☐ Krampfanfälle?____________

☐ Erste Auffälligkeiten in welchem Lebensalter?____________

Was?____________

☐ Sonstige Auffälligkeiten?____________

Kindergarten ☐ Probleme?____________

☐ Kontaktschwierigkeiten? Distanzlosigkeit?____________

Schule ☐ Klasse?____________

☐ Schwierigkeiten?____________

Pubertät ☐ ja ☐ nein

Auffälligkeiten?____________

Menstruation Beginn:____________ regelmäßig? ☐ ja ☐ nein

Sonnenverträglichkeit ☐ ja ☐ nein

Bisherige Untersuchungen

☐ Chromosomenanalyse? Molekulargenetische Diagnostik? Wo? Wann?

____

☐ Hörtest? Wo? Wann?

____

☐ Augenuntersuchung? Wo? Wann?

____

☐ Stoffwechseluntersuchungen? Wo? Wann?

____

☐ MRT (Schädel)? Wo? Wann?

____

☐ EEG? Wo? Wann?

____

☐ Röntgen linke Hand? Wo? Wann?

____

☐ Weiteres?

____

Körperlicher Untersuchungsbefund

Aktuelle Größe: ____________cm Vater: __________cm Mutter: _________cm

Kopfumfang: ____________cm Vater: __________cm Mutter: _________cm

Gewicht: ____________cm

Körperbau: ☐ proportioniert ☐ unproportioniert

☐ Adipositas? ☐ Asymmetrie?

Kopf: Haare, Haaransatz: ________________ Stirn: ________________

Augenstellung: ________________ Augenfarbe: ________________

Telekanthus/Hyper-/Hypotelorismus: _____ cm Proptosis? ____________

Ohren: ________________ Nase: ________________

Philtrum: ________________ Mund: ________________

Kinn: ________________ Gaumen: ________________

Sonstiges: ______________________________________

Hals: Pterygium? ______________________________________

Thorax/ Abdomen: Mamillenabstand: ______cm Trichter-/Hühnerbrust? ☐ ja ☐ nein

Skoliose? ☐ ja ☐ nein Nabelbruch? ☐ ja ☐ nein

Hände: ☐ unauffällig ☐ Klinodaktylie ☐ Brachydaktylie

Füße: ☐ unauffällig ☐ Syndaktylie Schuhgröße: __________cm

Nägel: ______________________________________

Genitale: ___________ Pubertätsstadien: ___________ Hodenhochstand? ☐ ja ☐ nein

Haut: ☐ Café-au-lait-Flecken? ☐ Pigmentveränderungen?

☐ Trockene Haut? ☐ White spots?

☐ Adenoma sebaceum? ☐ Sonstiges ________________
